# Offspring conceived through ART have normal thyroid function in adolescence and as young adults

**DOI:** 10.1093/humrep/deac095

**Published:** 2022-05-08

**Authors:** L A Wijs, D A Doherty, J A Keelan, V Panicker, P Burton, J L Yovich, R J Hart

**Affiliations:** Division of Obstetrics and Gynaecology, University of Western Australia, Perth, WA, Australia; Division of Obstetrics and Gynaecology, University of Western Australia, Perth, WA, Australia; Women and Infants Research Foundation, Perth, WA, Australia; Division of Obstetrics and Gynaecology, University of Western Australia, Perth, WA, Australia; Women and Infants Research Foundation, Perth, WA, Australia; University of Western Australia, School of Biomedical Sciences, Perth, WA, Australia; Department of Endocrinology, Sir Charles Gairdner Hospital, Perth, WA, Australia; Division of Internal Medicine, University of Western Australia, Perth, WA, Australia; School of Medical and Health Sciences, Edith Cowan University, Perth, WA, Australia; Concept Fertility Centre, Perth, WA, Australia; School of Pharmacy and Biomedical Sciences, Curtin University, Perth, WA, Australia; PIVET Medical Centre, Perth, WA, Australia; Division of Obstetrics and Gynaecology, University of Western Australia, Perth, WA, Australia; Fertility Specialists of Western Australia, Perth, WA, Australia

**Keywords:** ART, IVF, ICSI, thyroid function, long-term outcomes, ART outcomes, adolescents, young adults

## Abstract

**STUDY QUESTION:**

Are there differences in thyroid function between adolescents and young adults conceived with and without ART?

**SUMMARY ANSWER:**

This study demonstrated no evidence of clinically relevant differences in thyroid function between adolescents and young adults conceived with and without ART.

**WHAT IS KNOWN ALREADY:**

Studies to date have reported an increase in subclinical hypothyroidism in offspring conceived after ART. It has been suggested that the increase in maternal estrogen (E2) after fresh embryo transfers could affect thyroid function of the offspring. Suboptimal thyroid function at a young age can cause irreversible damage to the central nervous system, which makes early detection and correct treatment essential.

**STUDY DESIGN, SIZE, DURATION:**

The Growing Up Healthy Study (GUHS) is a prospective cohort study, which aimed to recruit all adolescents born after conception with ART between 1991 and 2001 in the study area. The included participants (n = 303, aged 13–20 years) completed various health assessments. Depending on the age at enrolment, participants completed thyroid assessments at the 14- or 20-year follow-up. The outcomes of these replicated thyroid assessments were compared to those of participants conceived without ART from the Raine Study Generation 2 (Gen2). The Gen2 participants (n = 2868) were born between 1989 and 1992 and have been recognized to be representative of the local population.

**PARTICIPANTS/MATERIALS, SETTING, METHODS:**

Thyroid function assessments were compared between n = 134 GUHS and n = 1359 Gen2 adolescents at age 14 years and between n = 47 GUHS and n = 914 Gen2 young adults at age 20 years. The following mean thyroid hormone concentrations were compared between the cohorts: thyroid-stimulating hormone (TSH), free triiodothyronine (fT3), free thyroxine (fT4) and thyroid peroxidase antibodies (TPOAb). The prevalence of the following thyroid hormone profiles, based on individual thyroid hormone concentrations, was compared: euthyroidism, subclinical and overt hypo- and hyperthyroidism and thyroid autoimmunity. Outcomes were compared between the cohorts, and univariately between fresh embryo transfers (ET) and frozen ET (FET) within the GUHS. The correlation between maternal peak E2 concentrations (pE2) and fT4 was assessed within the GUHS.

**MAIN RESULTS AND THE ROLE OF CHANCE:**

All mean thyroid function outcomes fell within the normal range. At both ages, we report no differences in TSH concentrations. At age 14 years, lower fT3 concentrations (4.80 versus 5.35 pmol/L, *P* < 0.001) and higher fT4 concentrations (12.76 versus 12.19 pmol/L, *P* < 0.001) were detected in the GUHS adolescents compared to Gen2 adolescents. At age 20 years, higher fT3 and fT4 concentrations were reported in GUHS adolescents (4.91 versus 4.63 pmol/L, *P* = 0.012; 13.43 versus 12.45 pmol/L, *P* < 0.001, respectively) compared to Gen2 participants. No differences in the prevalence of subclinical and overt hypo- and hyperthyroidism or thyroid autoimmunity were demonstrated between the cohorts at age 14 and 20 years. Thyroid function did not differ between ET and FET, and no correlation between pE2 and fT4 was reported.

**LIMITATIONS, REASONS FOR CAUTION:**

The observational nature of the study limits the ability to prove causation. Furthermore, the comparison of ET and FET offspring at age 20 years may be lacking power. We were unable to differentiate between different types of ART (e.g. IVF versus ICSI) owing to the low number of ICSI cycles at the time of study. As ART laboratory and clinic data were collected contemporaneously with the time of treatment, no other data pertaining to the ART cycles were sought retrospectively; hence, some factors could not be accounted for.

**WIDER IMPLICATIONS OF THE FINDINGS:**

This study does not support previous findings of clinically relevant differences in thyroid function when comparing a cohort of adolescents conceived after ART to counterparts conceived without ART. The minor differences detected in fT3 and fT4 were considered not biologically relevant. Although these findings appear reassuring, they warrant reinvestigation in adulthood.

**STUDY FUNDING/COMPETING INTERESTS:**

This project was funded by an NHMRC Grant (Hart *et al.*, ID 1042269). R.J.H. is the Medical Director of Fertility Specialists of Western Australia and a shareholder in Western IVF. He has received educational sponsorship from MSD, Merck-Serono and Ferring Pharmaceuticals. P.B. is the Scientific Director of Concept Fertility Centre, Subiaco, Western Australia. J.L.Y. is the Medical Director and a shareholder of PIVET Medical Centre, Perth, Western Australia.

**TRIAL REGISTRATION NUMBER:**

N/A.

## Introduction

ART, such as IVF and ICSI, have been used since 1978 to assist infertile couples to conceive. In Australia, currently, 1 in 20 children is born after assisted conception, while worldwide over 8 million children have been born after ART (Fauser, 2019; Newman *et al.*, 2020). Short-term health outcomes of offspring conceived after ART have been studied extensively and indicate an increased risk of preterm birth (Declercq *et al.*, 2015), low birthweight (LBW) (Schieve *et al.*, 2002), congenital malformations (Pandey *et al.*, 2012; Qin *et al.*, 2015) and imprinting disorders (Vermeiden and Bernardus, 2013). Increasingly, studies are now investigating longer term health outcomes in adolescents conceived after ART. One of the outcomes of particular interest is thyroid function, as this may be influenced by the large fluctuations in maternal estrogen (E2) concentrations around conception. Studies investigating thyroid function in offspring conceived after ART are relatively limited. However, all studies to date report evidence of differences in thyroid function between offspring conceived with and without ART. Studies have reported an increase in subclinical hypothyroidism (SCH) (Sakka *et al.*, 2009; Onal *et al.*, 2012), and an increase in free triiodothyronine (fT3) (Gkourogianni *et al.*, 2014), free thyroxine (fT4) and thyroid-stimulating hormone (TSH) concentrations in ART-conceived offspring (Lv *et al.*, 2014). It has been suggested that an increase in maternal E2 after fresh embryo transfers (ET) could affect thyroid function of the offspring through direct effects on the thyrocytes (thyroid epithelial cells) and through epigenetic alterations (Manole *et al.*, 2001; Banu *et al.*, 2002; Ho *et al.*, 2006). A Greek study, suggested that the reported increase in TSH in ART-conceived children aged 4–14 years may be related to an epigenetic alteration in the set point of TSH, resulting in mild TSH resistance (Sakka *et al.*, 2009). This is in line with previous studies showing that ART may induce epigenetic alterations around the susceptible window of conception, which can lead to the development of various disorders, including thyroid dysfunction, later in life (Fleming *et al.*, 2018).

Thyroid dysfunction at a young age can cause irreversible damage to the central nervous system, with subsequent mental impairment. Quality of life, anxiety, symptoms of depression, cognitive function and memory can be altered in patients with SCH, although studies are conflicting (Baldini *et al.*, 1997; Canaris *et al.*, 2000; Jorde *et al.*, 2006; Cooper and Biondi, 2012). In addition, changes in thyroid function can alter metabolic parameters such as lipid profiles, which, if left untreated, can lead to cardiovascular disease later in life (Duntas, 2002; Rodondi *et al.*, 2010; Delitala *et al.*, 2017). As most of these unfavourable effects appear to be reversible with the levothyroxine replacement therapy, early detection of thyroid dysfunction is essential for correct treatment (Serter *et al.*, 2004; Razvi *et al.*, 2007). If there is a significant association between ART conception and thyroid dysfunction, this opens possibilities for routine screening and subsequent treatment for ART-conceived offspring.

The aim of this study was to compare the thyroid function of a cohort of adolescents and young adults conceived after ART to that of a well established and representative cohort of similar age and sex, conceived without ART.

## Materials and methods

Thyroid function was compared between a total of 181 adolescents conceived after ART (n = 134 at age 14 and n = 47 at age 20 years) and 1359 adolescents conceived without ART (n = 1359 at age 14 and n = 914 at age 20 years).

### Study populations

#### ART cohort

**Figure 1.**
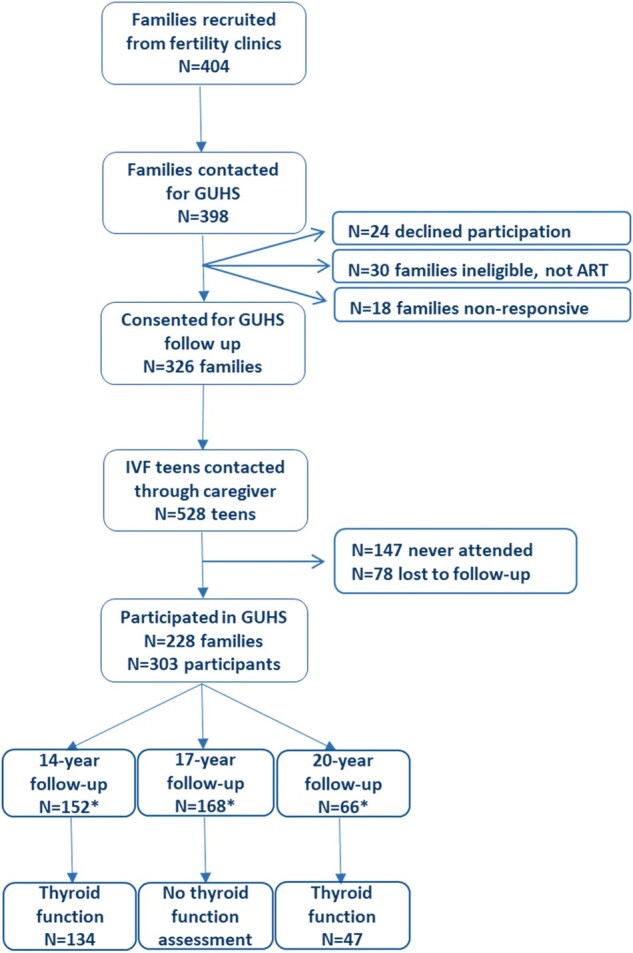
**Flowchart of study recruitment process of the Growing Up Healthy Study**. *Numbers do not total N = 303, as some participants completed >1 follow-up (depending on age at enrolment).

The Growing Up Healthy Study (GUHS) is a prospective cohort study investigating the long-term health of adolescents and young adults conceived after ART. The study aimed to recruit all adolescents conceived after ART in the two fertility clinics operating at the time (1991–2001): PIVET Medical Centre and Concept Fertility Centre, both in Perth, Western Australia, Australia. Four hundred and four families were recruited through the fertility clinics and a final number of 303 eligible adolescents participated in the study. Included adolescents completed various health assessments at ages 14, 17 and 20 years, which included thyroid function assessments at ages 14 and 20 years. Assessments were conducted between 2013 and 2017, at which time point n = 152 were 14 years of age and n = 66 were 20 years of age. Of these, n = 134 and n = 60 underwent thyroid function assessments. Thirteen offspring in the 20-year follow-up were conceived through gamete intra-fallopian transfer (GIFT) and were respectively excluded from the thyroid function analyses. This resulted in thyroid function assessment results of n = 134 at age 14 years, and n = 47 at age 20 years, used in this study. Numbers at the 20-year follow-up are lower owing to the infrequent use of ART in the early 1990s. The full recruitment process can be viewed in Fig. 1. Included participants were conceived through IVF (n = 125) and ICSI (n = 40), of whom n = 100 were fresh ETs and n = 65 frozen ETs (FET). Sixteen participants had a confirmed ART status but unconfirmed type of ART. owing to age ineligibility, none of the included GUHS participants completed thyroid assessments at both follow-ups. GUHS participants replicated assessments previously completed by adolescents of similar age and sex, conceived without ART, from the Raine Study. Outcomes of these health assessments were then compared between the cohorts.

#### Non-ART cohort

The Raine Study is a cohort study, which recruited 2900 pregnant women in Western Australia between 1989 and 1991 to investigate the safety and effects of ultrasound on the foetus (https://www.rainestudy.org.au) (Newnham *et al.*, 1991). A total of 2868 children born to 2804 mothers formed Raine Study Generation 2 (Gen2). The Gen2 participants have completed assessments at various follow-ups throughout their lives, to investigate the effect of perinatal health on subsequent childhood and adult health (Straker *et al.*, 2017). Assessments were completed at birth, at ages 1, 2, 3, 5, 8 and 10 years and then at ages 14, 17, 18, 20, 22, 27 and 28 years. The cohort (aged 30–31 years) has a current follow-up rate of 70%, including 1800 participants. The Raine study adolescents are representative of the Western Australian adolescent population at the time of assessments (Robinson *et al.*, 2010). The 14- and 20-year follow-ups, including thyroid function assessments, were completed by 1369 Gen2 participants (in 2003–2005 and 2009–2011, respectively). Overall, results from n = 1359 Gen2 participants conceived without ART (age 14 years n = 1359, and age 20 years n = 914) were included in this study, after the exclusion of five participants conceived after IVF and five participants conceived after GIFT.

### Data collection

#### Laboratory and thyroid function assessments

At both follow-ups, fasting blood samples for biochemical analyses were taken and plasma was frozen until TSH, fT3, fT4 and thyroid peroxidase antibodies (TPOAb) concentrations were measured by automated immunoassay using Abbott ARCHITECT analyser (Abbott Diagnostic, IL, USA). Mean TSH, fT3, fT4 and TPOAb concentrations were compared between the cohorts. Reference ranges, derived by local consensus and based on local population-based studies and laboratory data (O'Leary *et al.*, 2006; Panicker *et al.*, 2010), are: TSH 0.4–4.0 mU/L, fT3 3.0–6.5 pmol/L (age 11–14 years), 3.0–5.0 pmol/L (age 15–18 years), 3.0–5.5 pmol/L (age ≥19 years) and fT4 9–19 pmol/L. TPOAb positivity is defined as TPOAb >6 kU/mL (according to the manufacturer’s recommendation) at all ages. Thyroid hormone profiles were compared based on individual concentrations of thyroid function assessments: euthyroidism (TSH: 0.4–4.0 mU/L, fT4: 9–19 pmol/L, fT3 age 14 years: 3–6.5 pmol/L, fT3 age 19–20 years: 3–5.5 pmol/L), subclinical hypothyroidism (↑TSH, ↔fT4), hypothyroidism (↑TSH, ↓fT4), subclinical hyperthyroidism (↓TSH, ↔fT4), hyperthyroidism (↓TSH, ↑fT4), and autoimmune thyroid disorder (TPOAb >6 kU/L). Participants were classified as having autoimmune thyroid disorder if they had positive antibodies (TPOAb >6 kU/L) and did not fall into another category. The effect of maternal peak circulating peri-ovulatory estrogen (pE2) was examined by comparing thyroid function assessments of ETs and FETs separately to Gen2 and to each other within the GUHS. It was further examined whether there was a correlation between maternal pE2 and fT4 within the GUHS cohort (n = 160).

#### Anthropometric assessments

At both follow-ups, standing height was measured using a stadiometer (to the nearest 0.1 cm) and weight was measured seated, with participants lightly clothed, using an electronic scale (to the nearest 0.1 kg). BMI was calculated (kg/m^2^).

#### Questionnaires

Detailed questionnaires containing health and demographic information were used for information regarding smoking and use of medications in the past 6 months. At age 14 years, questionnaires were completed by the participant and the primary caregiver, and at age 20 years, the questionnaires were completed by the participant. Furthermore, for Gen2 participants information regarding the type of conception and pregnancy outcomes [i.e. gestational age (GA), birthweight (BW) and multiplicity] was collected via questionnaires.

#### Fertility clinic records

For all GUHS participants, information regarding cause of infertility, previous IVF cycles, IVF cycle of index pregnancy, medication scheme, day of ET, donor use, maternal peak circulating pE2 (in pmol/L) and pregnancy outcomes, was collected from the clinical records at PIVET Medical Centre and Concept Fertility Centre.

#### Midwives notification system

Additional information regarding pregnancy outcomes for GUHS pregnancies was collected from the Midwives Notification System from the Western Australian Department of Health. This was primarily used in the case of missing information from fertility clinics, as these clinics did not always hold information regarding pregnancy outcomes.

### Ethical approval

The study received ethical approval for both contacting the participants as well as collection of data from the University of Western Australia (RA/4/1/5860) and from the Human Research Ethics Committee of the Western Australian Department of Health (project number 2013/25). Informed and written consent for the study and for every follow-up was obtained from the parents or guardians for participants who were underage, or from the participants themselves if they were over the age of 18 years.

### Statistical analysis

Differences in demographic characteristics between groups were evaluated by Student’s *t*-test, Mann–Whitney *U* test, Chi^2^ test or Fisher’s exact test. For continuous outcomes univariate cohort, differences were examined using Mann–Whitney *U* tests and for categorical outcomes Chi^2^ and Fisher’s exact tests were used. Adjusted analyses were performed using generalized estimating equations that modelled siblings as a random factor. In addition to adjustment for sex, current age and BMI (Gkourogianni *et al.*, 2014; Lv *et al.*, 2014), further covariates were added to account for cohort differences that may also influence thyroid function (i.e. non-singleton, BW *z*-score, GA and use of anticonvulsants). Outcomes at both ages were adjusted for: non-singleton, BW *z*-score, GA, sex and age of participant. At age 20 years, analyses were additionally adjusted for BMI and use of anticonvulsants in the past 6 months, owing to differences between the cohorts on these covariates at age 20 years. Log transformations of thyroid hormone concentrations were conducted when normality assumption was not met, and the estimated back-transformed means and their 95%CI were reported. Effects of ART in the categorical models were summarised using adjusted odds ratios and their 95% CI. Stratifying analyses by sex did not alter the results, and to maintain statistical power, analyses for both sexes were combined. Univariate subgroup analyses were performed by comparing outcomes between ET and FET (age 14 years: n = 70 versus 53; age 20 years: n = 30 versus 12), and by comparing ET and FET separately to Gen2 participants. Spearman correlation was used to assess an association between pE2 and fT4 (n = 160). All analyses were performed in IBM SPSS Statistics version 25.0 (IBM Corp, Armonk, NY, USA). *P* < 0.05 were considered significant.

## Results

### Demographics

Table I gives an overview of demographic characteristics at ages 14 and 20 years. The GUHS cohort demonstrated a significantly lower mean birthweight (age 14 years: *P* < 0.001; age 20 years: *P* = 0.042), lower GA (age 14 and 20 years: *P* < 0.001), as well as significantly more multiple births at both ages (age 14 years: *P* < 0.001; age 20 years: *P* = 0.029) than the Gen2 participants. The GUHS cohort was significantly older at both follow-ups (age 14 years and age 20 years: *P* < 0.001). At age 20 years, GUHS adolescents had a significantly lower BMI (*P* = 0.017) and an increased use of anticonvulsants in the past 6 months, compared to Gen2 (*P* = 0.001). Smoking and use of other medication did not differ between the cohorts at age 20 years. BMI and the use of medication did not differ at age 14 years. Sex distribution did not differ statistically at either age, although apparently fewer GUHS males participated at age 20 years.

**Table I:** Demographic characteristics for the ART and non-ART cohorts at ages 14 and 20 years.

Demographics	Age 14 years		*P*-value	Age 20 years		*P*-value
	*ART	**Non-ART		*ART	**Non-ART	
	(N = 134)	(N = 1359)		(N = 47)	(N = 914)	
**Age** (years)	14.9 (14.3 to 15.6)	14.1 (14.0 to 14.2)	**<0.001**	20.4 (20.1 to 20.9)	19.9 (19.7 to 20.3)	**<0.001**
	[13.1 to 16.9]	[13.0 to 15.1]		[18.0 to 23.0]	[19.1 to 22.1]	
**Sex**						
Male	68 (50.7%)	705 (51.9%)	0.796	19 (40.4%)	487 (53.3%)	0.085
**Birthweight** (g)	3175 (2794 to 3616)	3375 (3020 to 3678)	**0.001**	3120 (2829 to 3555)	3375 (3033 to 3660)	**0.042**
	[970 to 4470]	[760 to 5550]		[1580 to 4360]	[845 to 5185]	
**Birthweight **(*z*-score)	−0.098 (−0.825 to 0.541)	−.076 (−0.527 to 0.590)	0.181	−0.256 (−0.634 to 0.430)	0.036 (−0.515 to 0.550)	0.176
	[−3.057 to 8.392]	[−2.454 to 3.741]		[−1.618 to 2.046]	[−2.294 to 3.396]	
**Birthweight** (g)						
<2500	**20 (14.9%)**	**115 (8.5%)**	**0.034**	**7 (15.9%)**	**69 (7.6%)**	0.133
2500–3999	**99 (73.9%)**	**1109 (81.7%)**		33 (75.0%)	761 (83.4%)	
>4000	15 (11.2%)	133 (9.8%)		4 (9.1%)	83 (9.1%)	
**Birth length** (cm)	49 (47 to 51)	49 (48 to 51)	0.709	49 (48 to 51)	49 (48 to 51)	0.681
	[34 to 56]	[33 to 57]		[41 to 53]	[35 to 57]	
**Gestational age** (weeks)	38.4 (37.4 to 39.4)	39.7 (38.3 to 40.6)	**<0.001**	38.7 (37.5 to 39.8)	39.4 (38.3 to 40.6)	**<0.001**
	[26.9 to 42.0]	[24.6 to 42.9]		[32.4 to 41.0]	[24.6 to 42.9]	
**Gestational age** (weeks)						
<37 weeks	28 (22.8%)	139 (10.3%)	<0.001	9 (21.4%)	92 (10.1%)	0.048
37–42 weeks	**95 (77.2%)**	**1192 (87.9%)**		33 (78.6%)	804 (88.1%)	
>42 weeks	0 (0.0%)	25 (1.8%)		0 (0.0%)	17 (1.9%)	
**Plurality**						
Singleton	**97 (72.4%)**	**1299 (95.7%)**	**< 0.001**	**41 (87.2%)**	**873 (95.5%)**	**0.029**
Twin	**34 (25.4%)**	**56 (4.1%)**		**6 (12.8%)**	**38 (4.2%)**	
Triplet	**3 (2.2%)**	**3 (0.2%)**		0 (0.0%)	3 (0.3%)	
**BMI** (kg/m^2^)	20.3 (18.5 to 22.7)	20.4 (18.6 to 23.2)	0.451	21.6 (19.7 to 25.2)	23.3 (21.3 to 26.2)	**0.017**
	[14.7 to 33.3]	[14.0 to 43.8]		[18.0 to 34.9]	[15.5 to 51.7]	
**Use of anticonvulsants** in past 6 months	1 (0.7%)	8 (0.6%)	0.469	4 (8.5%)	6 (0.7%)	**0.001**
**Use of oral steroids** in past 6 months	0 (0%)	4 (0.3%)	1.000	0 (0.0%)	0 (0.0%)	1.000
**Use of OCP females** in past 6 months	2 (3.1%)	16 (2.5%)	0.679	7 (28.0%)	210 (47.3%)	0.060
**Smoking**						
Yes	–	–		5 (10.6%)	109 (11.9%)	0.435

OCP, oral contraceptive pill. Data are presented as median (25th–75th percentile) [range] and as N (%). Differences in demographic characteristics between groups were evaluated by Student’s *t*-test, Mann–Whitney *U* test, Chi^2^ test or Fisher’s exact test. Bold text indicates statistical significance.

* The Growing Up Healthy Study is a prospective cohort study, which aimed to recruit all adolescents born after conception with ART between 1991 and 2001 in the study area.

** Participants conceived without ART from the Raine Study Generation 2 (Gen2). The Gen2 participants were born between 1989 and 1992 and have been recognised to be representative of the local population.

**Table II:** Estimated mean thyroid hormone concentrations (95% CI) at ages 14 and 20 years.

Thyroid function	Age 14 years						Age 20 years					
	*ART	**Non-ART	*P*-value univariate	ART	Non-ART	*P*-value adjusted^1^	*ART	**Non-ART	*P*-value univariate	ART	Non-ART	*P*-value adjusted^2^
TSH mU/L	2.22 (2.05–2.40)	2.07 (2.02–2.13)	0.115	2.03 (1.82–2.26)	1.94 (1.82–2.05)	0.401	1.89 (1.63–2.16)	2.34 (2.06–2.62)	0.185	1.84 (1.58–2.15)	1.91 (1.77–2.07)	0.581
fT3 pmol/L	5.12 (5.02–5.22)	5.48 (5.44–5.51)	**<0.001**	4.80 (4.61–4.98)	5.35 (5.27–5.42)	**<0.001**	4.89 (4.70–5.07)	4.68 (4.65–4.71)	**0.003**	4.91 (4.69–5.14)	4.63 (4.55–4.71)	**0.012**
fT4 pmol/L	13.10 (12.86–13.33)	12.30 (12.22–12.37)	**<0.001**	12.76 (12.50–13.06)	12.19 (12.02–12.36)	**<0.001**	13.53 (13.15–13.92)	12.59 (12.50–12.67)	**<0.001**	13.43 (12.97–13.93)	12.45 (12.27–12.65)	**<0.001**

fT3, free triiodothyronine; fT4, free thyroxine; TSH, thyroid-stimulating hormone. Comparison between ART versus non-ART: age 14 univariate: TSH n = 134 versus 1337, fT3 n = 134 versus 1352, fT4 n = 134 versus 1348, age 14 adjusted: TSH n = 123 versus 1333, fT3 n = 123 versus 1348, fT4 n = 123 versus 1344; age 20 univariate: TSH n = 47 versus 905, fT3 n = 47 versus 914, fT4 n = 47 versus 914, age 20 adjusted: TSH n = 38 versus 870, fT3 n = 38 versus 878, fT4 n = 38 versus 838. For continuous outcomes, univariate cohort differences were examined using Mann–Whitney *U* test and for categorical outcomes Chi^2^ and Fisher’s exact tests were used. Adjusted analyses were performed using generalized estimating equations that modelled siblings as a random factor. Bold text indicates statistical significance.

* The Growing Up Healthy Study is a prospective cohort study, which aimed to recruit all adolescents born after conception with ART between 1991 and 2001 in the study area.

** Participants conceived without ART from the Raine Study Generation 2 (Gen2). The Gen2 participants were born between 1989 and 1992 and have been recognised to be representative of the local population.

1 Adjusted for: non-singleton, birthweight *z*-score, gestational age, sex and age of participant.

2 Adjusted for: non-singleton, birthweight *z*-score, gestational age, sex, age and BMI of participant and use of anticonvulsants in the past 6 months.

### Thyroid function

#### 14-Year follow-up

At age 14 years, thyroid function was compared between n = 134 GUHS adolescents and n = 1359 Gen2 adolescents (Table II). Mean TSH concentrations did not differ between GUHS and Gen2 participants (*P* = 0.401) and dividing TSH into quartiles showed no difference (*P* = 0.532). Mean fT3 concentration was significantly lower in GUHS versus Gen2 participants in univariate and adjusted analyses (*P* < 0.001), while mean fT4 concentration was significantly higher in GUHS versus Gen2 participants in both univariate and adjusted analyses (*P* < 0.001). The proportion of participants with positive TPO antibodies did not differ between GUHS versus Gen2 participants (4.5% versus 4.7%, *P* = 0.927) (data not shown). The prevalence of the thyroid hormone profiles did not differ between the cohorts (e.g. euthyroidism, subclinical and overt hypothyroidism, subclinical and overt hyperthyroidism, and autoimmune thyroid disorder) (*P* = 0.374) (Supplementary Table SI). Comparing these parameters separately between ET, FET and offspring conceived without ART did not alter the results (*P* = 0.874) (data not shown). Mean pE2 concentrations were significantly higher in pregnancies after ET versus FET (6561.4 versus 1538.7 pmol/L, *P* < 0.001). Comparison of thyroid function between ET (n = 53) versus FET (n = 70) showed no differences in mean concentrations of TSH (*P* = 0.513), fT3 (*P* = 0.400) and fT4 (*P* = 0.171) (Supplementary Table SII). No correlation between pE2 and fT4 concentrations was detected (correlation coefficient = 0.106, *P* = 0.243). Comparing ET and FET separately to Gen2 showed the same trends as comparing the entire GUHS cohort to Gen2 (Supplementary Table SIII). Stratification by sex did not alter the results when comparing GUHS versus Gen2 (data not shown).

#### 20-Year follow-up

At age 20 years, thyroid function was compared between n = 47 GUHS young adults and n = 914 Gen2 young adults (Table II). Mean TSH concentrations did not differ between the cohorts (*P* = 0.581) and dividing TSH into quartiles did not show any differences (*P* = 0.457). Mean fT3 and fT4 concentrations were significantly higher in GUHS versus Gen2 participants (*P* = 0.012 and *P* < 0.001, respectively). The proportion of participants with positive TPO antibodies did not significantly differ between the cohorts (*P* = 0.203) (data not shown). The prevalence of the thyroid hormone profiles did not differ between the cohorts (e.g. euthyroidism, subclinical and overt hypothyroidism, subclinical and overt hyperthyroidism and autoimmune thyroid disorder) (*P* = 0.565) (Supplementary Table SI). Comparing these parameters separately between ET, FET and offspring conceived without ART did not alter the results (*P* = 0.985) (data not shown). Mean pE2 concentrations were significantly higher in pregnancies after ET versus FET (8388.3 versus 1762.9 pmol/L, *P* < 0.001). Comparison of thyroid function between ET (n = 12) versus FET (n = 30) showed no differences in mean concentrations of TSH (*P* = 0.989), fT3 (*P* = 0.288) and fT4 (*P* = 0.536) (Supplementary Table SII). No correlation between fT4 and pE2 was detected (correlation coefficient: 0.025, *P* = 0.883). Comparing ET and FET separately to Gen2, showed the same trends as comparing the entire GUHS cohort to Gen2 (Supplementary Table SIII). Stratification by sex did not alter the results when comparing GUHS versus Gen2 (data not shown).

## Discussion

In the present study of 14- and 20-year-old participants, we report no evidence of suboptimal thyroid function in those who were conceived after ART, when compared to those conceived without ART. All mean thyroid function assessments fell within the normal range at both ages and no differences in the percentage of participants with thyroid disorders were detected. However, fT3 and fT4 concentrations differed statistically at both follow-ups. As the absolute differences are small and fall within the normal range, they are not clinically significant. These findings are reassuring, although require replication and follow-up. A study by Sakka *et al.* (2009) suggests that the reported increase in SCH in 106 ART newborns could potentially be explained by epigenetic alterations in the set point of TSH sensitivity, resulting in a mild TSH resistance. In our current study, at age 20 years, TSH concentrations were comparable between the cohorts (minimally lower in the GUHS versus Gen2 participants, albeit non-significant) and fT3 and fT4 concentrations were significantly higher in the ART cohort, which could potentially indicate early differences in thyroid function towards SCH, and should be followed up in later adulthood.

Similar to the findings by Sakka *et al.* a Turkish study of ART newborns also reported an increase in TSH concentrations (n = 98) (Onal *et al.*, 2012). The authors suggest that this could be related to an increase in HCG concentrations in ART pregnancies (Heinonen *et al.*, 1996). HCG is a thyrotrophic hormone, meaning that it has a direct influence on the secretory activity of the thyroid gland. Studies have shown that thyroid function in women after repeated ART cycles was normal, but anti-TPO concentrations were high, potentially affecting foetal thyroid development (Yoshimura and Hershman, 1995; Bussen *et al.*, 2000). In our study, information on HCG concentrations was not available; however, we demonstrated no differences in thyroid function when comparing fresh ET with FET.

Studies have suggested an association between increased maternal E2 concentrations in fresh ET pregnancies and altered thyroid function, such as an increase in thyroid hormone concentrations, in the offspring (Mintziori *et al.*, 2011; Poppe *et al.*, 2011; Santin and Furlanetto, 2011; Gracia *et al.*, 2012). Suggested underlying mechanisms are alterations in foetal DNA methylation after E2 exposure, as well as a direct effect of E2 on thyrocytes (Manole *et al.*, 2001; Banu *et al.*, 2002; Ho *et al.*, 2006). In a 2014 study, researchers reported significantly increased concentrations of T4, fT4 and TSH in 3–10-year-old singletons born after fresh ET compared to offspring conceived without ART (total n = 949), as well as in a separate set of newborns (n = 183) (Lv *et al.*, 2014). A correlation between concentrations of fT4 in the ART newborns and maternal serum concentrations of E2 during early pregnancy was demonstrated. Thyroid function of children born after FET was closer to that of offspring conceived without ART. Interestingly, the authors demonstrated that E2 concentrations were significantly increased after fresh ET throughout the entire first trimester, and not just around time of implantation (Lv *et al.*, 2014).This is important, because the most critical period of foetal thyroid development is the first trimester of pregnancy. In the present study, no differences were detected in concentrations of TSH, fT3 and fT4 when comparing ET with FET although, particularly at the 20-year follow-up, our analyses were lacking statistical power due to low numbers. However, these findings are supported by the lack of a correlation between maximum maternal pE2 and fT4 concentrations in the GUHS offspring.

LBW, among other features, has been shown to characterize women who develop spontaneous hypothyroidism in adult life (Kajantie *et al.*, 2006). The increased risk of LBW in ART pregnancies might explain the increase in (subclinical) hypothyroidism reported by some studies. In our study, the ART cohort was born at a significantly lower birthweight and earlier gestation, which could mediate a difference in thyroid function, however adjusting the analyses for birthweight *z*-scores and GA did not alter the results. Studies have furthermore demonstrated an association between multiple gestations and congenital hypothyroidism, however whether this association also exists for acquired SCH is unclear (Olivieri *et al.*, 2007). In our study the ART cohort contained significantly more multiples, although adjusting the analyses for ‘non-singletons’ did not alter the results. A Greek study comparing thyroid function between 42 ICSI-conceived children and 42 controls (mean age 6.80 years), reported a significant increase in fT3 concentrations in ICSI offspring, with no differences in fT4 and TSH concentrations (Gkourogianni *et al.*, 2014). An increase in fT3 concentrations could be translated to an increased peripheral conversion of fT4 to fT3, which can be seen in obese patients (Ortega *et al.*, 2012). In the present study, fT3 concentrations were significantly increased in ART offspring at age 20 years, whereas BMI was significantly decreased, and adjustment for BMI did not alter the results. The numbers in our study did not allow for separate investigation of ICSI-conceived offspring. Lastly, iodine deficiency can alter thyroid function; however, at present, the Western Australian population appears to have a sufficient iodine status. In a 2003–2004 study, West Australian children were found to be iodine replete and, as of 2009, mandatory iodine fortification of bread was introduced. Therefore, we believe it can be assumed that the study participants were iodine sufficient, as all assessments in both cohorts were conducted after 2003 (Li *et al.*, 2006). Furthermore, as ART pregnancies are always planned, most women may be expected to have an adequate vitamin intake during early pregnancy.

Detection of suboptimal thyroid function is important, as untreated it can lead to serious and irreversible damage to the central nervous system and long-term unfavourable cardiovascular parameters (Lv *et al.*, 2014). Most unfavourable effects are reversible with correct treatment, which makes early detection essential (Serter *et al.*, 2004; Razvi *et al.*, 2007). Despite other studies reporting evidence of a suboptimal thyroid function in ART offspring, our study does not support these findings. This is the first study investigating thyroid function beyond adolescence. Potentially early life differences in thyroid function, caused by epigenetic alterations, are mitigated later in life and therefore no longer present during adolescence and beyond. A recent study demonstrated no differences in methylation profiles between the GUHS and Gen2 offspring (Penova-Veselinovic *et al.*, 2021). However, as mean concentrations of fT3 are significantly lower at age 14 years and higher at age 20 years, and mean fT4 concentrations are significantly higher at both ages in the ART cohort, albeit within the normal range but potentially indicating early signs of altered thyroid function, verification of our findings later in life, as well as in larger cohorts, is of great importance.

### Strengths and limitations

The main strength of this study is the study design, where the adolescents conceived after ART underwent the same assessments as previously completed by their representative counterparts conceived without ART. In combination with the relatively static population of Western Australia, this is a particular study design that would be difficult to replicate elsewhere in Australia or internationally. In addition, as it was attempted to approach all adolescents conceived after ART in Western Australia during a 10-year period, through the two operational fertility clinics at the time of study, selection bias is reduced. To our knowledge, this is the first study investigating thyroid function in ART-conceived offspring beyond childhood. The study also has various limitations: study size is limited, and therefore the results need to be interpreted with caution and replicated in independent cohorts. Furthermore, sample size did not allow for comparison of different types of ART (i.e. IVF and ICSI) or an adequately powered comparison of ET and FET at age 20 years. To maintain power the presented analyses are not stratified by sex. However, when running analyses differentiated by sex, the results were similar without any suggestion of a sex-specific effect. Furthermore, the study adjusted for the use of anticonvulsants at age 20 years, as this significantly differed between the cohorts. However, information regarding the specific type of anticonvulsant was not available and could not be accounted for. Similarly, it was not possible to adjust for use of thyroid hormone, growth hormone and aspirin. Prospective recruitment of the ART cohort during adolescence and young adulthood prevented the ability to adjust for maternal health and pregnancy complication data. No information regarding maternal thyroid disorders during pregnancy was available. Lastly, having adjusted for BW and GA may bias the total effect of ART on thyroid function, although this effect is likely minimal as adjusting for these factors did not alter the results.

## Conclusion

Our study does not support the previous findings (reported in 2012–2014) of clinically significant altered thyroid function in ART adolescents. The differences detected in fT3 and fT4 concentrations at both ages (14 and 20 years) are not clinically relevant at this age, but should be reinvestigated in adulthood. Our findings are reassuring although they require replication in independent, and preferably larger, cohorts. Knowledge about long-term health outcomes in ART offspring, such as thyroid function, is important to provide informed advice on any risks and guidance on the appropriate screening and treatment options.

## Data availability

The data underlying this article cannot be shared publicly for ethical reasons and privacy protection of the individuals who participated in the study. The data will be shared upon reasonable request to the corresponding author.

## Supplementary Material

deac095_Supplementary_Table_SI

deac095_Supplementary_Table_SII

deac095_Supplementary_Table_SIII
