# Supplementary material for: Offspring conceived through ART have normal thyroid function in adolescence and as young adults
Source: Hum Reprod. 2022 May 8;37(7):1572–80. doi: 10.1093/humrep/deac095 (PMC9308959; doi:10.1093/humrep/deac095)
Supplement: deac095_Supplementary_Table_SI [file deac095_supplementary_table_si.pdf]

Human Reproduction, pp. 1–1, 2022
https://doi.org/10.1093/humrep/deac095

human reproduction

SUPPLEMENTARY DATA

Supplementary Table SI **Comparison of thyroid hormone profiles between ages (14 and 20 years).**

Thyroid hormone profiles	Age 14 years			Age 20 years		
	ART	Non-ART	*P*-value	ART	Non-ART	*P*-value
Euthyroidism	119 (88.8%)	1230 (90.6%)		42 (89.4%)	803 (87.9%)	
Hypothyroidism	–	–		0 (0%)	3 (0.3%)	
Hyperthyroidism	0 (0.0%)	2 (0.1%)		0 (0%)	5 (0.5%)	
Subclinical hypothyroidism	9 (6.7%)	59 (4.3%)	0.374	1 (2.1%)	55 (6.0%)	0.565
Subclinical hyperthyroidism	0 (0.0%)	1 (0.1%)		–	–	
Autoimmune thyroid disorder*	6 (4.5%)	(3.3%)		4 (8.5%)	38 (4.2%)	
Unclassified (missing >1 value)	0 (0%)	22 (1.6%)		0 (0%)	10 (1.1%)	

Comparison between ART versus non-ART: age 14: n = 134 versus 1358; at age 20: n = 47 versus 914.
*Positive thyroid peroxidase antibodies >6 kU/L, and not falling into another category. Data are presented as N (%).
