# Supplementary material for: Offspring conceived through ART have normal thyroid function in adolescence and as young adults
Source: Hum Reprod. 2022 May 8;37(7):1572–80. doi: 10.1093/humrep/deac095 (PMC9308959; doi:10.1093/humrep/deac095)
Supplement: deac095_Supplementary_Table_SII [file deac095_supplementary_table_sii.pdf]

Human Reproduction, pp. 1–1, 2022
https://doi.org/10.1093/humrep/deac095

human reproduction

SUPPLEMENTARY DATA

Supplementary Table SII Fresh versus frozen embryo transfers within GUHS—estimated means concentrations and their 95% CIs.

Fresh ET versus FET	Age 14 years			Age 20 years		
	Fresh ET	FET	*P*-value univariate	Fresh ET	FET	*P*-value univariate
TSH mU/L	2.10 (1.85–2.34)	2.28 (2.02–2.54)	0.513	1.79 (1.41–2.18)	1.90 (1.54–2.26)	0.989
fT3 pmol/L	5.18 (5.03–5.33)	5.06 (4.91–5.21)	0.400	4.82 (4.34–5.31)	4.98 (4.79–5.17)	0.288
fT4 pmol/L	12.92 (12.55–13.29)	13.21 (12.91–13.53)	0.171	13.33 (12.46–14.20)	13.57 (13.09–14.04)	0.536

FET, frozen embryo transfer; fresh ET, fresh embryo transfer; fT3, free triiodothyronine; fT4, free thyroxine; GUHS, Growing Up Healthy Study; TSH, thyroid-stimulating hormone. Comparison between fresh ET versus ET: at age 14: n = 70 versus 53; at age 20: n = 30 versus 12.
