# Supplementary material for: Offspring conceived through ART have normal thyroid function in adolescence and as young adults
Source: Hum Reprod. 2022 May 8;37(7):1572–80. doi: 10.1093/humrep/deac095 (PMC9308959; doi:10.1093/humrep/deac095)
Supplement: deac095_Supplementary_Table_SIII [file deac095_supplementary_table_siii.pdf]

Human Reproduction, pp. 1–1, 2022
https://doi.org/10.1093/humrep/deac095

human reproduction

SUPPLEMENTARY DATA

Supplementary Table SIII **Fresh ET versus non-ART (Gen2) and FET versus non-ART (Gen2)—estimated mean concentrations and their 95% CIs.**

	Age 14 years			Age 20 years		
**Fresh ET versus non-ART**	**Fresh ET**	**Non-ART**	***P*-value univariate**	**Fresh ET**	**Non-ART**	***P*-value univariate**
TSH mU/L	2.10 (1.85–2.34)	2.07 (2.02–2.13)	0.700	1.79 (1.41–2.18)	2.34 (2.06–2.62)	0.443
fT3 pmol/L	5.18 (5.03–5.33)	5.48 (5.44–5.51)	**0.001**	4.82 (4.34–5.31)	4.68 (4.65–4.71)	0.455
fT4 pmol/L	12.92 (12.55–13.29)	12.30 (12.22–12.37)	**0.001**	13.33 (12.46–14.20)	12.59 (12.50–12.67)	0.084
**FET versus Gen2**	**FET**	**Non-ART**	***P*-value univariate**	**FET**	**Non-ART**	***P*-value univariate**
TSH mU/L	2.28 (2.02–2.54)	2.07 (2.02–2.13)	0.168	1.90 (1.54–2.26)	2.34 (2.06–2.62)	0.252
fT3 pmol/L	5.06 (4.91–5.21)	5.48 (5.44–5.51)	**<0.001**	4.98 (4.79–5.17)	4.68 (4.65–4.71)	**<0.001**
fT4 pmol/L	13.21 (12.91–13.53)	12.30 (12.22–12.37)	**<0.001**	13.57 (13.09–14.04)	12.59 (12.50–12.67)	**<0.001**

FET, frozen embryo transfer; fresh ET, fresh embryo transfer; fT3, free triiodothyronine, fT4, free thyroxine; Gen2, Raine Study Generation 2; TSH, thyroid-stimulating hormone. Comparison between fresh ET versus non-ART: age 14: TSH n = 70 versus 1337, fT3 n = 70 versus 1352, fT4 n = 70 versus 1348; age 20: TSH n = 30 versus 905, fT3 n = 30 versus 914, fT4 n = 30 versus 914. FET versus non-ART: age 14: TSH n = 53 versus 1337, fT3 n = 53 versus 1352, fT4 n = 53 versus 1348; age 20: TSH n = 12 versus 905, fT3 n = 12 versus 914, fT4 n = 12 versus 914. Bold indicates statistical significance.
